# Audiovisual Crossmodal Correspondence between Bubbles’ Size and Pouring Sounds’ Pitch in Carbonated Beverages

**DOI:** 10.3390/foods9080966

**Published:** 2020-07-22

**Authors:** Jérémy Roque, Jérémie Lafraire, Malika Auvray

**Affiliations:** 1 Breakthrough Innovation Group, Pernod Ricard, 75009 Paris, France; 2 Institut Paul Bocuse Research Center, 69130 Ecully, France; jeremie.lafraire@institutpaulbocuse.com; 3 CNRS, Institut des Systèmes Intelligents et de Robotique (ISIR), Sorbonne Université, 75005 Paris, France; auvray@isir.upmc.fr

**Keywords:** crossmodal correspondences, expectations, attention, beverages, carbonation, freshness perception

## Abstract

Visual and auditory carbonation have been separately documented as being two sensory markers of perceived freshness in beverages. The aim of the present study is to investigate the cross-modal interactions between these two dimensions of carbonation. Three experiments focused on crossmodal correspondences between bubble size and pouring sound pitch, which have never been investigated with ecological stimuli. Experiment 1, using an implicit association test (IAT), showed a crossmodal correspondence between bubble size and pouring sound pitch. Experiment 2 confirmed this pitch-size correspondence effect by means of a Go/No-Go Association Task (GNAT). Experiment 3 investigated the mutual dependence between pitch, size, and spatial elevation as well as the influence of attentional factors. No dependence was found, however pitch-size correspondences were obtained only in the condition requiring attentional processes, suggesting that these effects might be driven by top-down influences. These results highlight the robustness of the pitch-size crossmodal correspondence across stimulus contexts varying in complexity. Thus, this correspondence might be fruitfully used to modulate consumers’ perceptions and expectations about carbonated beverages.

## 1. Introduction

Due to the growing interest in the multisensory processes influencing eating and drinking behaviours (see [1,2,3,4,5,6] for reviews), the interactions between dimensions of the sensory signal occurring during food and beverage consumption have received a great deal of attention in different fields of research. In the case of flavour, several of these crossmodal interactions have already been reported (e.g., interactions between taste and smell [5] and vision and taste [7]). In addition, the relative contributions of each sensory modality to flavour perception have been partially documented. Some studies have recently provided empirical evidence on the influence of auditory cues on flavour perception (see [8] for a review) or other food and beverage dimensions. For instance, Zampini and Spence’s study [9] showed that a product’s sound properties (loudness, frequency) can modulate perceived crispness of potato chips. Indeed, this study showed that participants’ rating of both the crispness and staleness of potato chips was influenced by the loudness and/or the pitch of the auditory feedback elicited during the biting action.

Most researches have thus focused on the distinct influence of visual or auditory cues on flavour expectations or perception. Despite the fact that multisensory research applied to consumer experience is now a blossoming field of research, the audiovisual interactions occurring before consumption are still poorly documented. This is particularly surprising given that these interactions influence consumers’ subsequent experiences by triggering specific sensory expectations (see [7] for a review). In order to foster knowledge of the mechanism underlying the interactions between visual and auditory signals, an increasing number of studies investigated the mechanisms of crossmodal correspondences which correspond to a set of heterogeneous non-arbitrary associations between perceptual dimensions from different sensory modalities or higher-order dimensions (e.g., emotion, linguistic, see [10] for a review). The association between size and pitch is one of the most documented crossmodal correspondences. In particular, using simple cues such as pure tones and grey circles, it has been reported that smaller visually presented circles are typically matched with higher-pitched sounds and larger circles with lower-pitched ones [11,12,13].

In a review on multisensory integration, perception, and ecological validity, De Gelder and Bertelson [14] defended that although simple cues have been shown to elicit robust multisensory integration, it is reasonable to question whether simple cue combinations are sufficient to fully understand the complex naturalistic situations in which humans evolve. In line with this statement, recent studies have investigated crossmodal correspondences using more complex and ecological stimuli in the food and beverage domain. For instance, Velasco et al.’s study [15] showed crossmodal correspondences between the auditory properties of pouring water (lower pitch for hot water versus higher pitch for cold water) and the congruent words “hot drink” and “cold drink” (see also [16,17]). Spence and Gallace’s study [18] reported another crossmodal correspondence related to carbonation in beverages. In their study, carbonated water was more associated with high-pitched meaningless words, such as “kiki” and “takete”. By contrast, still mineral water was more strongly associated with lower-pitched pseudo-words, such as “bouba” and “maluma”.

Investigating crossmodal interactions between two sensory modalities already allows for bringing additional knowledge to research in flavor perception. However, it has also been reported that several crossmodal correspondences are mutually dependent [19]. According to Parise and Spence [19], it is likely that modifying one of the crossmodal correspondences will affect those that are mutually dependent. There is a necessity to investigate further the potential consequences of manipulating the perceptual features involved in many crossmodal correspondences, such as pitch, which may be part of a broader associative network. For instance, auditory pitch has correspondences not only with visual size but also with spatial elevation [11]. Visual stimuli with high elevation are associated with high-pitched tones and, conversely, stimuli with low elevation are associated with low-pitched tones. It should be noted that few studies have investigated cases of intramodal correspondences. One of them, reported by Evans and Treisman [11], actually failed to show an intramodal correspondence between size and spatial elevation. This can appear surprising since this correspondence has been found crossmodally (e.g., [20]). In addition, neither the size-elevation nor the pitch-elevation correspondences were investigated using complex and ecological stimuli.

Following from the literature described above, Experiments 1 and 2 reported here aim at investigating if there is an ecological counterpart of the pitch–size correspondence using more complex and ecological stimuli cuing carbonation in beverages. We hypothesize (1) that there are pitch-size crossmodal correspondences between small bubbles and high-pitched pouring sounds and between big bubbles and low-pitched pouring sounds; (2) that given the reported influence of audiovisual carbonation on perceived freshness in beverages [21], we hypothesize that a congruent semantic prime will improve participants’ performance in pitch-size crossmodal congruency tasks. Thus Experiment 3 aims at investigating the possible mutual dependence between pitch, size, and elevation using ecological stimuli cuing carbonation in beverages. In particular, we investigated whether a bi-dimensional congruent visual stimulus (e.g., small bubbles and high elevation in space) jointly presented with a congruent auditory stimulus (i.e., high pitch) improves participants’ performance.

## 2. Experiment 1: Pitch–Size Correspondence between Bubbles’ Size and Pouring Sounds’ Pitch

### 2.1. Methods

#### 2.1.1. Participants

Forty participants (50% female, mean age 48 ± 15 SD Standard Deviation) completed Experiment 1. They were recruited through the database of the Institut Paul Bocuse Research Center. All the participants reported normal or corrected-to-normal vision and audition. None of them had a particular expertise in beverages in terms of profession, education, or leisure activities. These two screening criteria were respected for the three reported experiments. Each individual session lasted approximately 45 min and the participants were compensated with a 15€ voucher. All the participants provided written informed consent prior to taking part in the study. The experiment was approved by the local ethics committee (University Hospital Center of Lyon, Lyon, France).

#### 2.1.2. Stimuli

**Figure 1:**
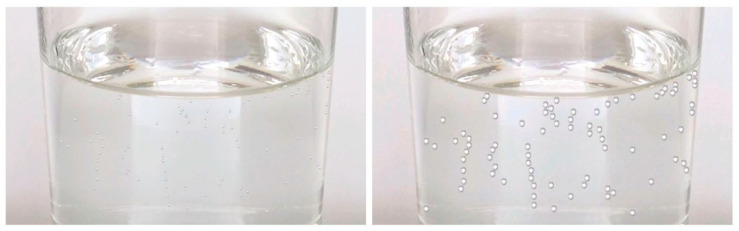
Pictures of the two visual stimuli with different bubbles’ sizes used in Experiment 1.

For the visual stimuli, pictures of 200 mL of colourless carbonated liquid (Schweppes Indian Tonic, 8.7 g/L CO_2_) were created. Pictures were taken in the same laboratory conditions in order to control for the light, room temperature, and ambient liquid temperature. All the pictures were taken when the liquid motion was stabilized and the foam was absent at the surface of the liquid (i.e., 2 s after pouring the 200 mL volume) since a negative effect of the foam on the participants’ RTs (reaction times) was found in previous research [21]. The picture in which the bubbles were the most visually identifiable was selected. Then, the picture’s background was standardized by keeping only one bubble using Microsoft’s application Paint (Version 1803). This bubble was copied and pasted on the locations of the other bubbles that were previously deleted. Finally, this picture was duplicated, and the bubbles’ sizes were modified to obtain four different sizes. Hence, four pictures were obtained, in which bubbles’ size was the only feature that varied. The lateral walls of the glass were kept within the picture to avoid any prototypical association of the shape of the glass with a particular drink (Figure 1).

For the auditory stimuli, the sound of 250 mL of liquid at ambient temperature (carbonated lemonade, 6.2 g/L CO_2_) poured into a glass was recorded for 2 Sec. Then, the background noise was reduced and four pouring sounds with different pitches (677 Hz, 886 Hz, 960 Hz, and 1086 Hz) were created using Audacity 2.1.3 software.

In order to select the optimal stimuli so that they are easily distinguishable one from the other, discriminability tasks were conducted with 6 participants (100% female, mean age 32 ± 3 SD). During the discriminability tasks (either visual or auditory), one picture (or one sound) was presented for 2 s, directly followed by the presentation of another picture or sound for 2 s. The participants’ task was to answer whether they perceived the bubbles’ size (or sounds’ pitch) of the second stimulus as smaller or bigger (higher or lower) as compared to the first stimulus (2 alternative forced choice). Error rates were calculated from the participants’ responses, which allow for determining the extent to which they were able to discriminate bubbles’ sizes and pouring sounds’ pitch. The visual stimuli that were better differentiated were those with the smallest and the third largest bubbles’ size (4.17% error rate), shown in Figure 1. The auditory stimuli that were better differentiated were the lowest (677 Hz) and the highest-pitched (1086 Hz) sounds (7.4% error rate).

#### 2.1.3. Design

A slightly modified version of the IAT (Implicit Association Test) described by Greenwald et al. [22] (see also Deroy et al. [23]) was programmed with E-prime^©^ (Psychology Software Tools, Version 2.0 Professional). This version incorporated the later suggestions for improvement made by Greenwald et al. [24], and it was also used in Crisinel and Spence’s study [25]. The first block of 24 trials consisted of practice at the bubbles’ size classification task. The second block of 24 trials consisted of practice at the pitch classification task. The third and fourth blocks consisted of the first combined task (16 and 48 trials, respectively), including the classification of both bubbles’ size and pouring sounds’ pitch. Half of the participants started with the same key for high-pitched sounds and small bubbles. For the other half of participants, the high-pitched sounds and big bubbles were initially associated with the same response key. The fifth block of 24 trials consisted of practice, this time for the small-big bubbles classification task with reversed response key associations. The sixth block consisted of the second (reversed) combined task.

As was suggested by Nosek et al. [26], the number of trials in this block was increased to 32 trials. The seventh and final block was made of 48 trials of the reversed combined task (see Table 1 for a summary of the IAT blocks). It should be noted that blocks three and six served as practice for blocks four and seven, respectively.

A semantic prime was included into the IAT task and the entire design was repeated twice in order to have one session with a neutral prime (neutral consonant letter string: “NGHTKLPRD,” see [27]) and another session with a prime related to freshness (i.e., “FRAICHEUR” in French). The two sessions were counterbalanced across participants and they were separated by a 10 min break. The participants completed 432 trials in total.

#### 2.1.4. Procedure

The experiment was conducted in a quiet testing room. The participants sat on a chair 70 cm from a liquid-crystal display (LCD) computer monitor with a resolution of 1600 × 900 pixels (60 Hz refresh rate). They wore headphones (Sony MDR-ZX110) for which the volume was adjusted individually to a clearly audible level. The participants’ level of thirst was evaluated twice on a 7-point Likert scale, once at the beginning of each session (neutral prime and freshness prime session). Before starting each session, the participants were also asked to drink a 200 mL glass of still water in order to control for their level of thirst (see [28] on the influence of thirst on alertness and cognitive performance).

The participants were instructed to categorize as rapidly and accurately as possible the visual and auditory stimuli by pressing one of the two response keys (D or J) on the computer keyboard with their left and right index fingers. Emphasis was put primarily on rapidity over accuracy; however, the participants were instructed to also try and avoid errors as much as possible. Instructions about the mapping between the stimuli and the relevant response keys consisted of a schematic representation of the two response keys with the corresponding stimuli that were displayed on the screen. There was no time limit to learn the new stimulus–response mapping that remained in written form at the top-right and top-left corners of the screen as a reminder throughout each block of the experiment. In each trial, the participants started by looking at a fixation cross at the centre of the screen for 1 s. Then, the semantic prime (either freshness-related prime or neutral prime according to the session) was displayed for 250 ms (as suggested by Wentura and Degner [27]), directly followed by the 2 s presentation of the target stimulus. Feedback, consisting of a red cross, was provided after each incorrect target-response and remained on the screen for 500 ms. Each trial was separated by a blank screen corresponding to the inter-trial stimulus interval (ISI) of 1 s. Participants’ RTs and accuracy were recorded.

At the end of the experiment, the participants were asked to rate which bubble’s size and which pouring sound’s pitch (without any particular stimuli presented) they generally associate with the consumption of fresh sparkling beverages on two 7-point Likert scales ranging from “Very small bubbles” to “Very big bubbles” and from “Very low-pitched sound” to “Very high-pitched sound”.

#### 2.1.5. Data Analysis

Five participants (2 males, 3 females) appeared as outliers in the data distribution and were removed from the subsequent analyses as they had more than 10% of unanswered trials (mean of trials unanswered: 21.4%; mean unanswered trials for the remaining thirty-five participants: 2.5%). The improved scoring algorithm suggested by Greenwald et al. [24] was used. Thus, trials from the third and sixth blocks, previously designed as practice, were included in the data analysis. In order to normalize RT distributions, RT data were log-transformed. In order to measure the IAT effect, D-measures were also calculated as effect-size measures from the participants’ RTs. D-measures were computed as the difference between mean RTs for blocks three and six (Mean block 6–Mean block 3) and blocks four and seven (Mean block 7–Mean block 4), for which each resulting difference was divided by the pooled standard deviation of the two corresponding blocks. Note that the computation of the D-measures was reversed for half of the participants, for whom these blocks were reversed. Moreover, RTs from the trials in which the participants responded correctly were submitted to a repeated-measure analysis of variance (ANOVA) with the within-participants factors of Congruency (Congruent associations: small bubbles + high-pitch, big bubbles + low-pitch; Incongruent associations: small bubbles + low-pitch, big bubbles + high-pitch), Modality (visual vs. auditory), Stimuli (small vs. big bubbles, low-pitched vs. high-pitched sounds), and Prime (neutral vs. freshness). The same analysis was performed considering the participants’ mean error rates (%) as the dependent variable. Tukey post-hoc analyses were subsequently performed. All the statistical analyses in this manuscript were performed using R 3.5.0., and the effects were considered significant when *p* < 0.05.

### 2.2. Results and Discussion

**Figure 2:**
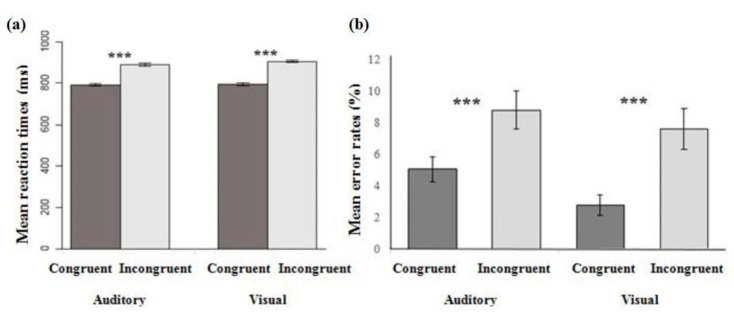
Mean reaction times (RTs) (**a**) and mean error rates (**b**) in response to the visual and auditory stimuli as a function of the congruency of the interaction. Error bars represent the standard error of the mean across participants and the asterisks indicate significant statistical differences (*p* < 0.001).

When considering the participants’ RTs as the dependent variable, the repeated-measure ANOVA revealed a significant main effect of Congruency (*F* = 481, *p* < 0.0001, partial η^2^ = 0.05) and Stimuli (*F* = 11.1, *p* < 0.0001, partial η^2^ = 0.001). The participants responded more rapidly in the congruent blocks of trials (m = 794 ms ± 3.9 standard error of the mean SEM) than in the incongruent ones (m = 897 ms ± 4.6 SEM), and this occurred independently of the modality (Figure 2a). Unlike previous studies on audiovisual crossmodal correspondences, in our study RTs for the visual stimuli were not shorter than those for the auditory stimuli [13]. This can be partly explained by the complexity of the stimuli used in our study compared to the quite simple and well-studied stimuli usually used in previous research. The presentation time of the targeted stimuli is also generally much shorter (300 ms) than the 2 s time-out used here, which could have influenced the stimuli’s processing time and thus impacted participants’ RTs.

When considering the mean error rates as the dependent variable, the repeated-measure ANOVA revealed only a significant main effect of Congruency (*F* = 20.7, *p* < 0.0001, partial η^2^ = 0.017). The participants made fewer errors in congruent blocks than in incongruent ones, independently of the modality (Figure 2b).

Regarding the significant effect of the stimuli, a Tukey post-hoc analysis revealed that the participants responded more rapidly to low-pitched sounds (m = 822.4 ms ± 5.7 SEM) than to high-pitched ones (m = 858.1 ms ± 6.6 SEM), *p* < 0.001. The interaction with Congruency was not significant (*F* = 1.1, *p* = 0.29). A Spearman correlation between the participants’ age and their RTs revealed that the older the participants, the slower they performed the task (rho = 0.29, *p* < 0.0001). This result is consistent with previous findings in cognitive science which reported that RTs are generally slower for elderly people than for younger people (e.g., [29]). The participants’ age did not significantly influence their mean error rates (*F* = 3.8, *p* = 0.061).

Finally, there was no effect of the prime on the participants’ RTs (*F* = 1.01, *p* = 0.3) or mean error rates (*F* = 0.8, *p* = 0.38). There was no significant trimodal interaction between the variables Congruency, Stimuli, and Prime on the participants’ RTs (*F* = 1.2, *p* = 0.3) or their mean error rates (*F* = 0.1, *p* = 0.8). Several hypotheses can explain the null effect of the prime, which have already been discussed in the literature on semantic priming. For instance, Klauer et al. [30] stated that even when a semantic congruency effect is expected (i.e., an overlap between prime-words and target stimuli), there is still uncertainty regarding the size of the overlap. Indeed, it is not certain that the overlap will be larger for congruent prime-target pairs than for incongruent ones. Whether any semantic overlap between the freshness prime and the target stimuli used in our study is large enough to cause a semantic congruency effect remains to be investigated. The ambiguous and heterogeneous meaning of freshness among people [31,32,33] might have reduced the ability to obtain semantic congruency effects when the word “freshness” was used as a semantic prime. Regarding response recordings, studies have shown that the response window procedure can help controlling speed-accuracy trade-off effects by reducing variances in the response latencies [30].

Regarding explicit measures, the results showed that the more the participants associated the consumption of fresh sparkling beverages with small bubbles, the more they also associated it with high-pitched pouring sounds (Spearman correlation: rho = 0.15, *p* < 0.0001). In particular, the data distribution for the pouring sounds’ pitch ratings shows that the participants associate the consumption of fresh sparkling beverages with very high-pitched sounds more than with very low-pitched ones.

Due to the null effect of the prime, for the computation of the D-measures described above, the participants’ RTs were combined across prime conditions. A one-sample t-test was conducted on the D-measures. The mean value was significantly different from zero ((Mean block 6–Mean block 3)/pooled standard deviation (sd) = 0.57 and (Mean block 7–Mean block 4)/pooled sd = 0.55; mean M = 0.56, SD = 0.46; t_34_= 7.15, *p* < 0.0001). This result confirms that the association between small bubbles and high-pitched sounds and the one between big bubbles and low-pitched sounds was stronger than the reverse associations. However, IAT designs do not allow for disentangling the relative effects of the two associations involved in the pitch–size correspondence (small bubbles–high pitch vs. big bubbles–low pitch). Indeed, when the participants respond to one stimulus (e.g., small bubbles) on the wrong response key, it is impossible to determine if the error is due to a wrong categorization or to an inhibition of the other visual stimulus (i.e., big bubbles). As a consequence, the evaluation of the stimuli is always relative [34]. Thus, the question of whether the crossmodal correspondence effect comes from the two associations or just one of them remains open and it is the question investigated in Experiment 2.

## 3. Experiment 2: The Robustness of the Pitch–Size Correspondence Effects

### 3.1. Methods

#### 3.1.1. Participants

Thirty participants (50% female, mean age 36 ± 15 SD) completed Experiment 2. The participants were recruited with the same criteria as in Experiment 1. The study also lasted approximately 45 min and the participants were compensated with a 15€ voucher.

#### 3.1.2. Stimuli

**Figure 3:**
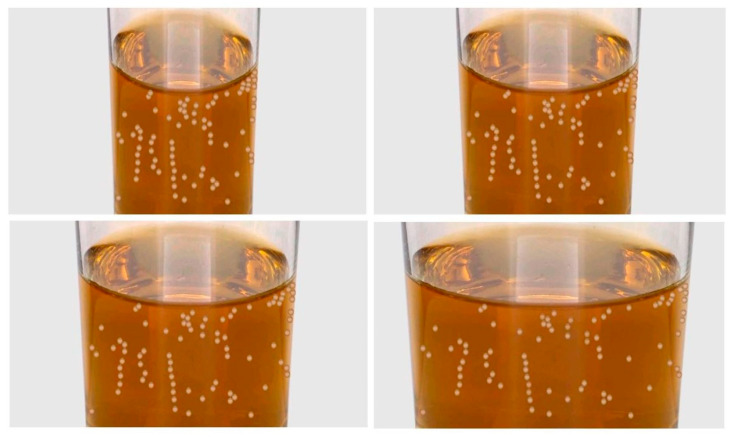
Pictures of the big bubbles visual stimuli used in Experiment 2, varying in width of the glass, for the brown liquid. See the Supplementary Materials Tables S1–S5 for the other sets of visual stimuli that were used.

In order to obtain a reliable GNAT (Go/No-Go Association Task) design for the analysis of the sensitivity in the participants’ responses, more stimuli than in Experiment 1 had to be introduced here. Thirty-six visual stimuli (one set of 12 stimuli for each bubble condition: small bubbles, big bubbles, and neutral stimuli) were created for this experiment. The neutral stimuli varied in the presence or absence of ice cubes and turbidity, but they did not contain any bubbles. These neutral stimuli were not relevant for the task; they were introduced to increase its difficulty. Within a set of 12 visual stimuli, there were 3 colours of the liquid (colourless, yellow, and brown), and 4 widths of the glass. In order to avoid fatigue and to keep the duration of the experiment to 45 min, only two colours of liquid, in addition to the colourless one, were selected based on the literature on freshness perception in beverages. In particular, yellow has often been reported as a freshness enhancer in beverages [33,35,36], whereas brown has most of the time been reported as a freshness inhibitor [33,36] even though there is no consensus on this effect [35]. For the colourless liquid stimuli, the same two as those of Experiment 1 were used here. The yellow and brown visual stimuli were created with 200 mL of still water to which food colourings were added (Figure 3). Bubbles for the yellow and brown stimuli were copied and pasted from the colourless stimuli and the colour contrast was adjusted using Photoshop. A preliminary discriminability task confirmed that variations in the colour of the beverage and the width of the glass did not significantly affect the perceived bubbles’ size (N = 8, 7 females, mean age 30 ± 10 SD; colour: *F* = 0.28, *p* = 0.76; width: *F* = 0.17, *p* = 0.92) nor the perceived bubbles’ quantity (*N* = 8, 5 females/3 males, mean age 24 ± 5 SD; colour: *F* = 1.23, *p* = 0.3; width: *F* = 2.5, *p* = 0.07).

The auditory stimuli were the same as in Experiment 1, with two additional pitches, one lower (576 Hz) and one higher (1187 Hz). These two pitches were added to increase task difficulty while still ensuring that they would be easily distinguishable one from the other. Thus, 4 auditory stimuli varying in pitch were used in Experiment 2: low pitch 1: 576 Hz, low pitch 2: 677 HZ, high pitch 1: 1086 Hz, and high pitch 2: 1187 Hz. Neutral auditory stimuli were not included in order to keep the duration of the experiment short. In summary, there were 4 different auditory stimuli and 36 different visual stimuli.

#### 3.1.3. Design

A slightly modified version of the GNAT described in Nosek and Banaji [37] was programmed with E-prime^©^ (Psychology Software Tools, Version 2.0 Professional), incorporating the later suggestions for improvement made by Williams and Kaufmann [38] for the appropriate number of trials.

The experiment consisted in 4 unimodal practice blocks (2 visual and 2 auditory) and four combined tasks (2 congruent and 2 incongruent). The practice blocks were made of 24 unimodal trials each. In the visual practice block, 8 random stimuli from each of the 3 sets of 12 visual stimuli (small bubbles, big bubbles, and neutral stimuli) were presented. In the auditory practice blocks, four pouring sound pitches were presented 6 times each in a random order. The order of the four practice blocks was randomized across participants.

Each of the four combined tasks was made of 24 practice trials and 72 experimental trials (critical combined task). Two blocks consisted in congruent associations (small bubbles + high-pitch, big bubbles + low-pitch) and two blocks consisted in the incongruent ones (small bubbles + low-pitch, big bubbles + high-pitch). In the 24 practice trials, 12 visual stimuli (4 random stimuli from each of the 3 sets of 12 visual stimuli) were presented as well as 12 auditory stimuli (2 high-pitched sounds and 2 low-pitched ones presented 3 times each). During the practice blocks, feedback was given on the correctness of the participants’ responses: a green circle or a red cross was presented on the screen for 100 ms, corresponding to a correct or an incorrect response, respectively. In the 72 experimental trials, there were 36 visual stimuli (each possible stimulus was presented once) and 36 auditory stimuli (2 high-pitched sounds and 2 low-pitched ones, presented 9 times each). This led to 72 trials involving 30 Go trials, 42 No-Go, and 12 task-irrelevant trials with the neutral visual stimuli. The four blocks of combined tasks were presented in a random order across participants. In total, each participant completed 480 trials (see Supplementary Materials Table S5 for a summary of the GNAT blocks).

#### 3.1.4. Procedure

The experiment was conducted in the same conditions as in Experiment 1. On-screen instructions were given before each block of trials. The participants were instructed to press the space bar as rapidly and accurately as possible if the image or the sound belonged to one of two target categories (e.g., small bubbles or high pitch). As a reminder to the participants, the target categories were presented at the top-left and top-right of the screen throughout the block. Each time, the participants had to press the space bar (Go) when one of the two stimuli involved in the association was presented or else refrain from responding (i.e., do not press the space bar, No Go). For instance, in the small bubbles + high-pitch condition, the participants had to press the space bar whenever they were presented with either small bubbles or a high-pitched sound. Trials were separated by an inter-stimulus interval (ISI) of 150 ms. Each stimulus (image or sound) was presented for 2 s or until the space bar was pressed. Participants’ RTs and accuracy were recorded. Emphasis was put on rapidity over accuracy; however, the participants were instructed to try and avoid errors as much as possible. The participants’ level of thirst was evaluated at the beginning of the experiment on a 7-point Likert scale. The participants were also asked to drink a 200 mL glass of still water in order to control for their level of thirst.

#### 3.1.5. Data Analysis

Two participants (1 male, 1 female) appeared as outliers in the data distribution and were removed from the subsequent analyses, due to more than 25% of their trials being unanswered (mean of trials unanswered: 27.4%; mean of trials unanswered for the remaining twenty-eight participants: 1.3%). To test for the influence of Congruency (congruent vs. incongruent), Modality (visual vs. auditory), Stimuli characteristics (bubble size, colour, width of the glass, pitch, pitch height), and Participants’ characteristics (gender, age, explicit freshness ratings of bubble size and pouring sound pitch) on the participants’ RTs, linear mixed-effects models were fitted to the data with participants as a random variable.

d’ was computed for each combined task according to Grier’s formulas [39]:

(1) d’=1/2+[(y − x)(1+ y − x)/4y(1− x)]

where y stands for the probability of a hit and x corresponds to the probability of a false alarm. d’ may thus range from 0 to 1, with 0.50 indicating responses at chance level, and 1 indicating maximum discriminability. Paired-sample t-tests were conducted to compare the participants’ d’ between blocks of trials in which the bubbles’ sizes were combined with high-pitched vs. low-pitched sounds.

### 3.2. Results and Discussion

When considering the participants’ RTs as the dependent variable, the final model (R^2^_C_ = 0.29, see [40]) revealed a significant main effect of Congruency (χ2(1) = 17.6, *p* < 0.001), Modality (χ2(2) = 9.5, *p* = 0.002), Bubbles’ size (χ2(3) = 14.6, *p* < 0.001), Colour of the liquid (χ2(5) = 24.3, *p* < 0.001), and Pitch height (χ2(8) = 29, *p* < 0.001). Overall, the participants’ RTs were significantly shorter in the congruent blocks (m = 704 ms ± 6.3 SEM) than the incongruent ones (m = 736 ms ± 6.6 SEM). This result is in line with those obtained in Experiment 1 and it confirms the existence of a pitch–size crossmodal correspondence. The participants’ RTs were also shorter for auditory stimuli (m = 716 ms ± 6.1 SEM) than for visual stimuli (m = 726 ms ± 7 SEM). The interaction between Modality and Congruency was not significant (*p* = 0.08), even if a tendency was observed between the mean RTs for visual stimuli in congruent blocks (m = 700 ms ± 9 SEM) as opposed to incongruent blocks (m = 752 ms ± 11 SEM).

A Tukey post-hoc analysis revealed that the participants’ RTs were shorter for low pitch 1 (576 Hz, m = 668 ms ± 9 SEM) than for low pitch 2 (677 HZ, m = 744 ms ± 11.6 SEM, *p* < 0.001) and high pitch 1 (1086 Hz, m = 729 ms ± 13.4 SEM, *p* = 0.0149). The participants also responded more rapidly to high pitch 2 (1187 Hz, m = 724 ms ± 13.7 SEM, *p* = 0.04) than to low pitch 2 (677 HZ). The interaction between pitch height and Congruency was not significant (*p* = 0.24). Thus, the pitch–size crossmodal correspondence occurred in all pitch height conditions.

The model also revealed a significant two-way interaction effect between colours of the liquid and bubbles’ size (χ2(10) = 10.3, *p* = 0.006). A Tukey post-hoc analysis revealed that the participants’ RTs were longer (*p* < 0.001) for the small bubbles when the beverage was yellow than for any other combination (small bubbles with colourless or brown beverages, and big bubbles with colourless, yellow, or brown beverages). The interactions between Congruency and the colour of the liquid (*p* = 0.97) and between Congruency and bubbles’ size (*p* = 0.29) were not significant.

An analysis of sensitivity did not show any significant effect of Congruency, Colour of the liquid, or Width of the glass (all *p* > 0.05). Here again, nothing can be concluded about the relative effects of the two associations involved in the pitch–size correspondence effect. Overall, the participants’ mean error rates were lower than 4%. Thus, the lack of differences between the two associations can be due either to an equal strength of the two associations in our experiment and/or to a task that might be too easy to perform for the participants to allow for showing any differences in sensitivity (d’).

## 4. Experiment 3: The Mutual Dependence of Crossmodal Correspondences and Attentional Effects

Experiment 3 aims at replicating the results reported in Experiments 1 and 2, and at bringing additional evidence on the robustness of the pitch–size correspondence effects between bubbles’ size and pouring sounds’ pitch. In addition, Experiment 3 aims at investigating the mutual dependence between pitch–size correspondence and spatial elevation, as well as attentional effects. This was done while taking into account the complex naturalistic situations that humans are facing during their drinking experiences.

### 4.1. Methods

#### 4.1.1. Participants

Forty-eight French participants took part in Experiment 3 (24 in Experiment 3a: 50% female, mean age 36 ± 13 SD; and 24 in Experiment 3b: 50% female, mean age 34 ± 11 SD). The participants were recruited with the same criteria as in Experiments 1 and 2. In Experiment 3a, each individual session lasted approximately 45 min and the participants received a 10 € voucher to complete the study. In Experiment 3b, each individual session lasted approximately 1 h and the participants received a 15 € voucher to complete the study. All the participants provided written informed consent prior to taking part in the study. The experiment was approved by the local ethics committee (University Hospital Center of Lyon).

#### 4.1.2. Stimuli

The visual stimuli were the same as in Experiment 1, with an additional picture involving an intermediate bubble size. This picture was selected from the discriminability task conducted before Experiment 1 (see Supplementary Materials Figure S1). The auditory stimuli were the same as in Experiment 1 (low pitch: 677 HZ, high pitch: 1086 Hz).

#### 4.1.3. Design and Procedure

Three crossmodal correspondences (pitch–size, pitch–elevation, and pitch–size–elevation) and one intramodal correspondence (size–elevation) were investigated. This was done across two types of tasks: the “direct tasks” in Experiment 3a and the “indirect tasks” in Experiment 3b (see details below). The experiments were conducted in a quiet testing room. The participants sat on a chair 70 cm from a LCD computer monitor with a resolution of 1600 × 900 pixels (60 Hz refresh rate). They wore headphones (Sony MDR-ZX110) for which the volume was adjusted to a clearly audible level.

For each tested correspondence, three conditions were investigated. One of them was unimodal and the other two were bimodal (or bi-dimensional); one consisting of a congruent and the other of an incongruent combination of stimuli. Each time, the participants were instructed to focus on one dimension of the signal (i.e., bubble’s size, pitch, or elevation) and to respond only to the stimulus presented in this dimension while ignoring the stimuli presented in the other dimensions. The participants were instructed to press the space bar as rapidly and accurately as possible if the picture or the sound belonged to target category. Emphasis was put on rapidity over accuracy, although the participants were instructed to try and avoid errors as much as possible. The visual and auditory stimuli were presented for 2 s or until the space bar was pressed. Trials were separated by an inter-stimulus interval (ISI) of 150 ms. On-screen instructions were given before each block of trials.

The participants’ level of thirst was evaluated at the beginning of each experiment on a 7-point Likert scale. Before starting each experimental session, the participants were also asked to drink a 200 mL glass of still water in order to control for their level of thirst.

##### Experiment 3a: Direct Tasks

In the direct tasks, the participants were asked to respond to the same features on which the correspondences were tested (i.e., bubbles’ size, pouring sounds’ pitch, and spatial elevation). Thus, for the pitch–size correspondence, the participants were asked to perform a speeded classification task according to pouring sounds’ pitch (high pitch vs. low pitch) and bubbles’ size (small bubbles vs. big bubbles). For the pitch–elevation correspondence, the participants had to classify pouring sounds’ pitch (high pitch vs. low pitch) and the spatial elevation of the visual stimuli (high elevation vs. low elevation with a fixed intermediate bubbles’ size picture. Note that this intermediate size was used to have one constant size. The size–elevation correspondence tested the speeded classification of bubbles’ size (small bubbles vs. big bubbles) and the spatial elevation of visual stimuli (high elevation vs. low elevation). Finally, the pitch–size–elevation correspondence tested the interaction between these three features. In each experiment, one-third of the trials consisted in unimodal presentations of the stimuli for comparison.

For both the pitch–size and pitch–elevation correspondences, there were 80 unimodal trials (40 visual and 40 auditory) and 80 bimodal trials. The size–elevation unidimensional task on spatial elevation contained 40 trials whereas the bidimensional tasks contained 80 trials. Finally, the bimodal and bidimensional tasks for the pitch–size–elevation correspondence contained 320 trials, giving rise to 760 trials in total. The number of trials was determined in order to have ten repetitions of each stimulus in the unimodal tasks and five repetitions of each stimulus in the bimodal tasks per condition. The number of targeted features within the stimuli was two in the unimodal tasks and four in the bimodal tasks. In the direct tasks, the location of the visual stimuli could be either central (for the pitch–size correspondence), low, or high elevation (for the size–elevation and pitch–size–elevation correspondences).

##### Experiment 3b: Indirect Tasks

In the indirect tasks, the participants were asked to respond to different features than those corresponding to the tested correspondences (i.e., bubbles’ size, pouring sounds’ pitch, and spatial elevation). In particular, for the pitch–size correspondence, the participants were asked to perform the speeded classification task either according to the lateralization of the auditory stimuli (sounds heard in their left ear versus right ear), or the lateralization of the visual stimuli (pictures seen on the right or left side of the screen). As in the direct tasks, the sounds varied in pitch and the pictures still varied in bubbles’ size. For the pitch–elevation correspondence, the participants also had to classify either the lateralization of both the auditory (still varying in pitch) and visual stimuli (fixed intermediate bubbles’ size). When responding to the auditory stimuli, the intermediate bubbles’ size picture was presented at two possible locations: high or low. When responding to the visual stimuli, there were four possible locations for the intermediate bubbles’ size picture: high elevation right/left vs. low elevation right/left. The size–elevation correspondence tested the speeded classification of pictures’ location. There were four possible locations: high elevation right/left vs. low elevation right/left. The pictures still varied in bubbles’ size. Finally, the pitch–size–elevation correspondence tested the interaction between all of these three features. In all the conditions, one-third of the trials were presented as unimodal stimuli for comparison.

For both the pitch–size and the pitch–elevation correspondences, the unimodal auditory and visual tasks were made of 80 trials each, and the bimodal tasks contained 160 trials. For size–elevation, both the unidimensional condition on the horizontal position and the bidimensional condition contained 80 trials. Finally, the bimodal and bidimensional conditions for the pitch–size–elevation correspondence included 320 trials giving rise to 1120 trials in total. The number of trials was determined according to the same criteria as in the direct tasks.

At the end of the two experiments (direct and indirect tasks), explicit measures about the association between bubbles’ size and pouring sounds’ pitch were collected. The participants were asked to indicate which bubbles’ size and which pouring sounds’ pitch (without any particular stimuli presented) they generally associate to the consumption of fresh sparkling beverages on two 7-point Likert scales ranging from “Very small bubbles” to “Very big bubbles” and from “Very low-pitched sound” to “Very high-pitched sound”.

#### 4.1.4. Data Analysis

In Experiment 3a, data from one participant were not analysed due to a software malfunction during the test. In Experiment 3b, six participants (2 males, 4 females) appeared as outliers in the data distribution and were removed from the subsequent analyses due to more than 40% of unanswered trials (mean 48.8%; mean of unanswered trials for the remaining eighteen participants: 3.2%). In order to normalize the RT distributions, the RT data were log-transformed. The RTs from those trials in which the participants responded correctly were submitted to mixed model analyses of variances with the within-participant factors of Correspondence (pitch–size, pitch–elevation, size–elevation, pitch–size–elevation), Congruency (congruent, incongruent, and unimodal/unidimensional) for each correspondence, Attended modality (target modality, to which the participants were asked to focus on for a given set of trials; visual vs. auditory), and the between-participant factor task (direct vs. indirect). The same analysis was performed considering the participants’ mean percentage of errors as the dependent variable. Tukey post-hoc analyses were subsequently conducted.

### 4.2. Results and Discussion

When considering the participants’ RTs as the dependent variable, the mixed model ANOVA revealed significant main effects of Task (*F* = 9.2, *p* = 0.004, partial η^2^ = 0.19), Correspondence (*F* = 16.5, *p* < 0.0001, partial η^2^ = 0.005), Pitch–Size Congruency (*F* = 11.8, *p* < 0.0001, partial η^2^ = 0.0015), Size–Elevation Congruency (*F* = 3.7, *p* = 0.02, partial η^2^ = 0.0005), and Attended Modality (*F* = 102, *p* < 0.0001, partial η^2^ = 0.006). There were significant interactions between Task and Correspondence (*F* = 25.3, *p* < 0.0001, partial η^2^ = 0.008) and between Task and Pitch–Size Congruency (*F* = 3.7, *p* = 0.03, partial η^2^ = 0.0005).

**Figure 4:**
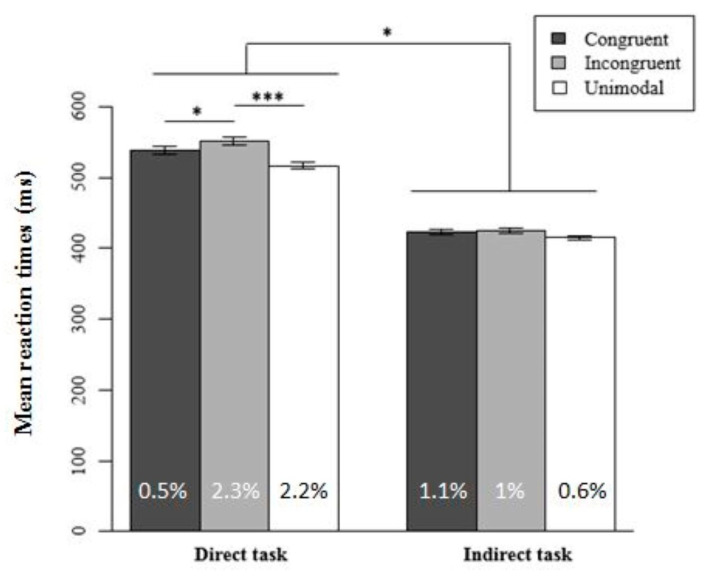
Mean reaction times as a function of the task and the congruency of the interaction for the pitch–size correspondence. Direct task: *N* = 23. Indirect task: *N* = 18. The mean percentage of errors for each condition is shown at the bottom of each column. Error bars represent the standard error of the mean across participants and the asterisks indicate significant statistical difference (* *p* < 0.05, *** *p* < 0.001).

Overall, the participants’ RTs were shorter in the indirect task (m = 417 ms ± 1.7 SEM) than in the direct one (m = 531 ms ± 2.6 SEM). Regarding the two-way interaction between Task and Pitch–Size Congruency, a Tukey post-hoc analysis revealed that the participants’ RTs were shorter in congruent trials (m = 537 ms ± 5.3 SEM) than in incongruent ones (m = 551 ms ± 5.5 SEM), in the direct task only (Figure 4), *p* = 0.02. However, there were no significant differences in mean RTs between congruent and incongruent trials according to the Attended Modality (visual vs. auditory), and this was the case independently of the task (direct vs. indirect). Therefore, in the direct task, there was an overall effect of pitch-size. As in Experiment 2, this effect did not occur for each sensory modality. The mean percentages of errors were not significantly different between the two conditions of Pitch–Size Congruency neither in the direct task nor in the indirect one. Even though further investigation is needed, our results suggest that pitch–size correspondence effects occur only when the participants’ attention is directed toward the same features on which the correspondence is tested (i.e., bubbles’ size versus pouring sounds’ pitch). In the direct task, the visual and auditory stimuli that the participants had to discriminate were presented in the task’s instructions. It is thus possible that during the processing of these stimuli along the task, the participants formed higher order associations between the target stimuli and the corresponding category to which they might relate (e.g., freshness perception in beverages, or positively correlated psychophysiological concept such as thirst-quenching, see [21,32]). According to this interpretation of our results, the pitch–size correspondence effects reported in the case of beverages might be driven by top-down influences such as attentional processes.

A Tukey post-hoc analysis conducted on size-elevation congruency revealed that the participants’ RTs were shorter in unidimensional trials (m = 516 ms ± 3.8 SEM) than in congruent bidimensional trials (m = 542 ms ± 6.2 SEM). However, there were no significant differences in mean RTs between bidimensional congruent and incongruent trials (*p* = 0.7). This null effect of the size–elevation correspondence is consistent with the results obtained by Evans and Treisman [11]. Our result brings additional evidence that the correspondence between size and elevation is actually not straightforward and further research is needed to confirm or infirm this relation. We believe that the complexity and the ecological character of the stimuli used here might require adjustments and additional testing. For instance, spatial elevation could be manipulated with bubbles in motion in the liquid instead of manipulating the position of the picture on the screen. However, Parise [19] underlined that while the use of complex stimuli might be more ecological, it is likely that the multidimensionality of such stimuli will increase the difficulty for researchers to identify what are the relevant stimulus dimensions that drive the corresponding crossmodal correspondences.

Regarding the interaction between Pitch, Size, and Elevation, the mixed model ANOVA revealed a significant effect of Congruency on the participants’ RTs only in the direct task (*F* = 3, *p* = 0.03, partial η^2^ = 0.002). The participants’ RTs for congruent pitch–size bimodal stimuli (m = 527 ms ± 8.2 SEM) were not significantly different from their RTs for congruent pitch–size–elevation bimodal stimuli (m = 542 ms ± 8.8 SEM). Thus, there is no additive facilitation effect since a bidimensional congruent visual stimulus (e.g., small bubbles and high elevation in space) presented together with a congruent auditory stimulus (i.e., high pitch) did not significantly reduce the participants’ RTs.

Finally, regarding the analysis of explicit measures, it appeared that the more the participants associated the consumption of fresh sparkling beverages with small bubbles, the more they also associated it with high-pitched pouring sounds (Spearman correlation: rho = 0.21, *p* < 0.0001).

## 5. General Discussion

The three experiments reported here aimed at investigating crossmodal correspondences between audiovisual stimuli cuing carbonation in beverages using more complex and ecological stimuli than those generally used in the literature on audiovisual correspondences [13]. More specifically, Experiment 1 aimed at testing if there are pitch–size correspondence effects between bubbles’ size and pouring sounds’ pitch and at investigating whether these effects can be modulated by a congruent semantic prime (IAT paradigm). Experiment 2 aimed at investigating the relative effects of the two associations involved in the pitch–size correspondence, previously uncovered in the first experiment. In addition, variations in the stimuli (colour of the liquid, width of the glass, and pitch) were included in order to test the robustness of the pitch–size correspondence effects (GNAT paradigm). Finally, Experiments 3a and 3b first aimed at testing the interactions between pitch and size on the one hand and spatial elevation on the other hand. The second aim was to investigate the influence of attentional factors on the correspondence effects (speeded classification task).

In Experiment 1, the results revealed shorter RTs and more accurate responses in congruent blocks than in incongruent ones. These results represent the first piece of empirical evidence showing a more ecological counterpart of the reported pitch–size correspondence. In particular, it reveals pitch–size correspondence effects between bubbles’ size and pouring sounds’ pitch. Inter-individual differences may play a role in the way participants indirectly associate the stimuli. For instance, McEwan and Colwill [41] reported that the level of carbonation required in a drink for it to be considered as either thirst-quenching or acceptable varies inter-individually. Although some people like a highly carbonated drink, most of them prefer a drink to be slightly sparkling or still. The number of bubbles in our experiments did not vary, but it is possible that the participants have associated a quantity of bubbles with a given size, to a specific carbonation intensity in a drink, and consequently to a certain valence.

Experiment 1 also investigated whether the semantic relation between small bubbles and high-pitched pouring sounds is more strongly related to the concept of freshness than the association between big bubbles and low-pitched pouring sounds, and whether this relation could influence participants’ performance. The results did not show any influence of the semantic prime on participants’ responses. The concept of freshness is broad and heterogeneous among people, even when it is used within the category of beverages [21,31,32,42]. Roque et al. [21,32] previously described the different meanings of freshness, which can refer to: (a) the overall multisensory experience during drinking (involving for instance coldness, sourness, or a menthol odour that will contribute to an actual perception of freshness), (b) the aging of the organic ingredients contained in the drink (e.g., aging of the mint leaves in a mojito), or else (c) the time delay (informed and/or perceived) to which the drink has been prepared before being served. As a consequence, this semantic ambiguity may prevent observing congruency effects when the word “freshness” is used as a semantic prime in relation to specific perceptual features of the beverage because only one meaning is relevant. Heyman et al. [43] reported that priming effects arise as a result of automatic pre-activation processes and/or the use of strategies such as expectancy generation and semantic matching. As a consequence, automatic priming emerges when the presentation of a related prime (partially) activates the target’s representation, thereby lowering its recognition threshold. Different theories and models are still debated in the literature in marketing [44] and in cognitive psychology [45] regarding the appropriate prime and target sizes, the familiarity of both primes and stimuli, or else the influence of personal characteristics such as individuals’ awareness, motivation, and capacity for evaluation.

In Experiment 2, the RT analysis confirmed the existence of the pitch–size correspondence effects revealed in Experiment 1. Variations in the colour of the liquid, width of the glass, and pitch did not significantly interact with the congruency of the audiovisual interaction. This suggests that the pitch–size correspondence effect is robust to variations in the stimulus context. The sensitivity in participants’ responses was influenced neither by the congruency of the interaction, the colour of the liquid, the width of the glass, nor by the pitch’s height. On the basis of our results, it is not possible to conclude on the relative effects of the two associations involved in the pitch–size correspondence reported in the IAT and the GNAT experiments.

Similar to the IAT experiment, the carbonation intensity was kept constant all along the GNAT experiment. It is possible that this fixed bubbles’ density, together with variations in the colour of the liquid, created a perceptual mismatch between the expected versus the actual attributes of the stimuli during the task. For instance, if one person expects brown carbonated drinks to have a high bubble density, then the discrepancy with the actual bubble density in our stimuli might interfere with the relation of congruency between the visual stimuli and the auditory stimuli.

In Experiment 3a (i.e., direct task), pitch–size correspondence effects were obtained. This result is consistent with those obtained in Experiments 1 and 2. Moreover, changes in participants’ attention allowed providing the first empirical evidence that the pitch–size correspondence effect is more likely to occur when participants’ attention is directed toward the same features on which the correspondence is tested (i.e., bubbles’ size and pouring sounds’ pitch). Two different modes of attention have been distinguished, namely the exogenous and the endogenous [46]. In Experiment 3, it is possible that the presentation of the stimuli only in the instructions of the direct task activated both endogenous and exogenous attentional processes toward the perceptual features of interest during the task, when the participants actually had to classify the previously presented stimuli. Consequently, these attentional processes could be partly responsible for the pitch–size correspondence effects that occurred in the direct task only.

In Experiments 3a and 3b, the results did not reveal any pitch–elevation nor size–elevation correspondence effects. The pitch–elevation correspondence investigated in Experiment 3 has generally been considered as semantically mediated since common terms are used to describe both pitch and elevation, namely high and low. However, it is worth underlining here that this dichotomy does not exist in French common language in which the verbal labels used to describe pitch and elevation are different (i.e., “aigu” and “grave” for pitch, “haut” and “bas” for elevation). Such lack of semantic mediation might explain the lack of pitch–elevation correspondence effects in Experiment 3. Against this hypothesis, several cross-cultural (e.g., [47]) and developmental (e.g., [48]) research have shown that the pitch–elevation correspondence was not purely semantic in nature as infants and individuals from cultures who use other terms to describe pitch still show effects of pitch directionality on elevation judgments. Cultural studies would enable verifying whether or not the pitch–elevation correspondence is culturally mediated as a function of the stimulus context.

The implicit measures collected in our experiments did not allow for determining whether the two associations (small bubbles–high pitch and big bubbles–low pitch) equally contribute to the global effect. However, when the participants were required in explicit questionnaires to assess which bubbles’ size and pouring sounds’ pitch they generally associate with fresh sparkling beverages, their results indicate that they associate small bubbles and high-pitched pouring sounds to freshness in beverages more than big bubbles and low-pitched pouring sounds. Several interpretations about the links between the results obtained with implicit and explicit measures have been proposed in the literature. If there is no implicit-explicit correlation, one of the possible interpretations is that the implicit measures have not been “contaminated” by the inherent biases that belong to the explicit measures (such as self-observation bias or limited introspective abilities). In this case, the dissociation between implicit and explicit measures can be interpreted as an index of discriminant validity. However, if there is an implicit–explicit correlation, researchers may interpret it as an index of convergent validity showing the reliability of the paradigm they used. In one of the reviews focusing on the IAT and related tasks, Teige Mocigemba et al. [49] reported that one ongoing debate is whether low (implicit–explicit correlation of 0.24, see [50]) to moderate (implicit–explicit correlation of 0.37, see [51]) correlations between the IAT and explicit measures should be interpreted as cues of discriminant or convergent validity. For instance, a self-observation bias can occur in explicit measures. However, a bias that corresponds to what the participants accept to say about their own perception, attitudes, or behaviours is unlikely to fully explain the dissociations between implicit and explicit measures in a crossmodal version of the IAT. In particular, in our study the participants had no reasons to hide or to be ashamed of a particular behaviour concerning the bubbles’ size and pouring sounds’ pitch that they associate to the consumption of fresh sparkling beverages. Correlational analyses between implicit and explicit measures and the computation of other cues showing the reliability of the used paradigm represent promising research avenues in the food and beverage domain. In particular, the combination of approaches may enable researchers to provide a more in-depth comprehensive model of consumers’ perception and behaviours, for instance in the case of complex multisensory perception such as freshness in beverages.

## 6. Conclusions

The results of the three experiments reported here confirm the existence of a pitch–size crossmodal correspondence between bubble size and pouring sound pitch in carbonated beverages. Experiment 1 provides the first empirical evidence that complex and ecological audiovisual stimuli also induce the pitch–size correspondence effects well-established with simple stimuli. The result obtained in Experiment 2 using GNAT did not allow for determining the relative effects of the two associations involved in the pitch–size crossmodal correspondence. However, such pitch–size crossmodal correspondence appears to be robust in several respects because the effects still hold when the colour of the liquid, width of the glass, and pitch varied. In Experiment 3, changes in participants’ attention showed that the pitch–size correspondence effect in beverages is more likely to occur when the participants’ attention is directed toward the same features on which the correspondence is tested (i.e., bubbles’ size and pouring sounds’ pitch). On the other hand, the results obtained with explicit measures suggest that the participants associate small bubbles and high-pitched pouring sounds with freshness in beverages more than big bubbles and low-pitched pouring sounds. In terms of applications, the results obtained for the pitch–size correspondence, both implicitly and explicitly, suggest that companies aiming at increasing beverage attractiveness and perceived freshness of their products would benefit from directing consumers’ attention toward the features of interest, such as the bubbles. This could be done, for instance, by increasing the saliency of the perceptual features associated with bubbles’ size and pouring sounds’ pitch. Understanding the relations between different crossmodal correspondences would represent a promising way to develop efficient strategies (in terms of formulation, packaging, retail experience, or ads) to better catch consumers’ attention and likely increase attractiveness and appreciation of products.

## Figures and Tables

**Table 1:** Summary of the design for the Implicit Association Test (IAT) blocks.

Block	Task	Left Key Response	Right Key Response	No of Trials
1	Single task (practice)	Small bubbles	Big bubbles	24
2	Single task (practice)	High-pitched	Low-pitched	24
3	Combined task	Small bubbles + high-pitched	Big bubbles + low-pitched	16
4	Combined task	Small bubbles + high-pitched	Big bubbles + low-pitched	48
5	Reversed single task (practice)	Big bubbles	Small bubbles	24
6	Reversed combined task	Big bubbles + high-pitched	Small bubbles + low-pitched	32
7	Reversed combined task	Big bubbles + high-pitched	Small bubbles + low-pitched	48

## Supplementary Materials

The following are available online at https://www.mdpi.com/2304-8158/9/8/966/s1, Table S1: References and volumes of food-coloring used to generate the yellow and brown visual stimuli in Experiment 2, Table S2: Pictures of the visual stimuli with small bubbles used in Experiment 2, Table S3: Pictures of the visual stimuli with big bubbles used in Experiment 2, Table S4: Pictures of the neutral visual stimuli used in Experiment 2, Table S5: Summary of the design for the GNAT blocks used in Experiment 2, Figure S1: Pictures of the three visual stimuli used in Experiments 3a and 3b, with different bubbles’ sizes.
