# Supplementary material for: Audiovisual Crossmodal Correspondence between Bubbles’ Size and Pouring Sounds’ Pitch in Carbonated Beverages
Source: Foods. 2020 Jul 22;9(8):966. doi: 10.3390/foods9080966 (PMC7466368; doi:10.3390/foods9080966)
Supplement: Supplementary file 1 [file foods-09-00966-s001.pdf]

## Supplementary Materials

**Experiment 2:**

**Table S1.** References and volumes of food-coloring used to generate the yellow and brown visual stimuli in Experiment 2

| **Color** | **Food-Coloring References** | **Food-Coloring Volume (μL)** | | | |
|---|---|---|---|---|---|
| | | **No Ice Cubes** | **4 Ice Cubes** | **No Ice Cubes + Turbidity** (cloudy agent: Symrise Ref 357255=10 μL) | **4 Ice Cubes + Turbidity** (cloudy agent: Symrise Ref 357255=6.6 μL) |
| **Yellow** | E104 | 200 | 132 | 240 | 158.4 |
| **Brown** | E150d | 50 | 33 | 50 | 33 |

The final volume in each condition was completed to 200 mL with still water at ambient temperature. The bubbles were subsequently added using Photoshop.

**Table S2.** Pictures of the visual stimuli with small bubbles used in Experiment 2

| | Colorless | Yellow | Brown |
|---|---|---|---|
| Width 1 | 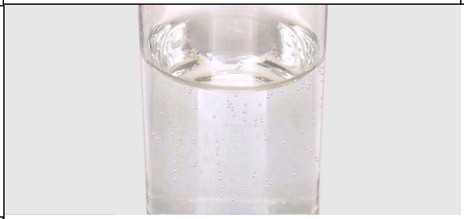 | 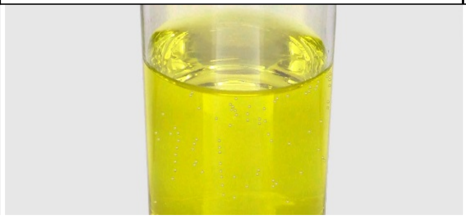 | 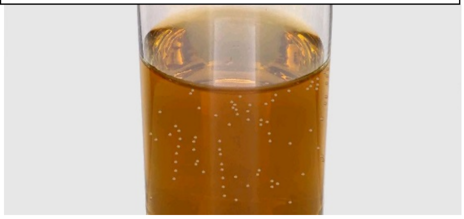 |
| Width 2 | 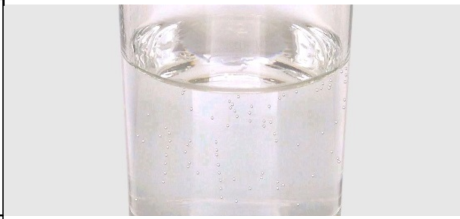 | 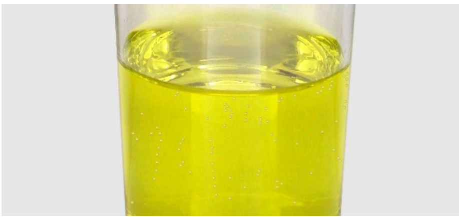 | 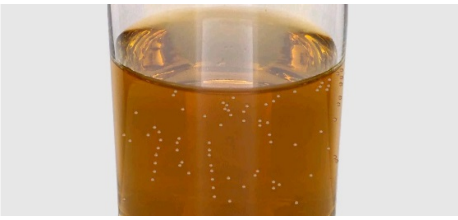 |
| Width 3 | 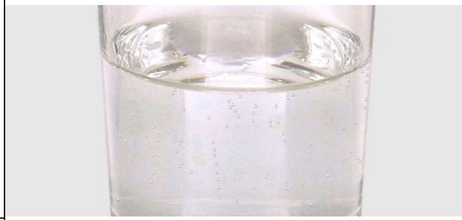 | 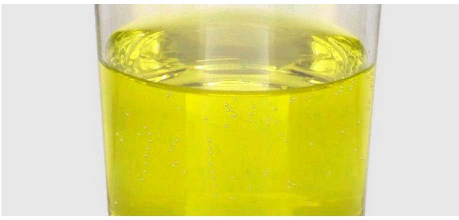 | 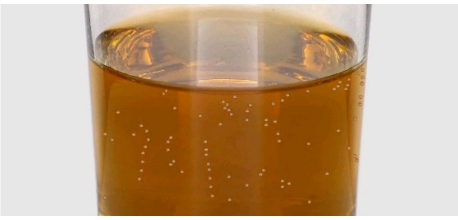 |
| Width 4 | 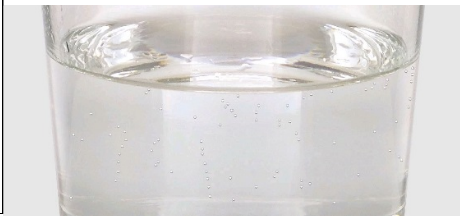 | 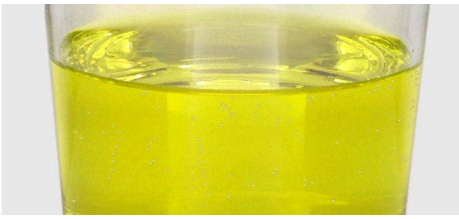 | 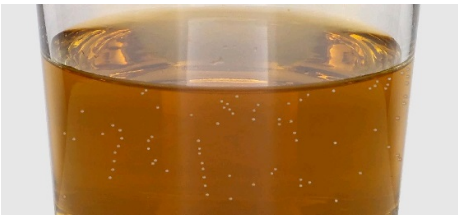 |

**Table S3.** Pictures of the visual stimuli with big bubbles used in Experiment 2

| | Colorless | Yellow | Brown |
|---|---|---|---|
| Width 1 | 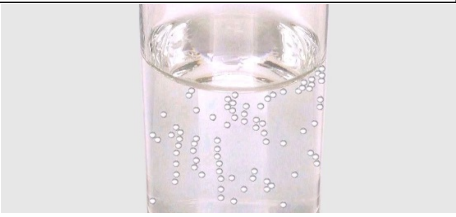 | 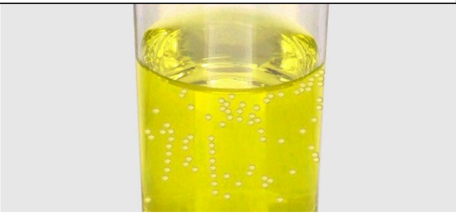 | 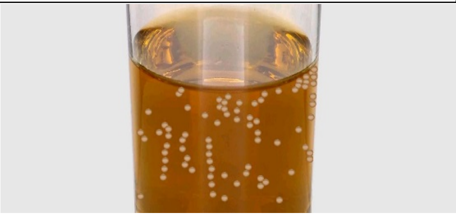 |
| Width 2 | 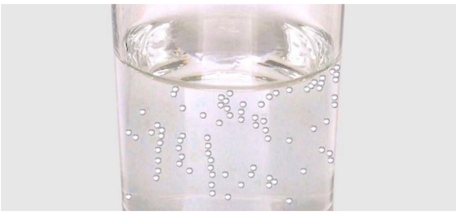 | 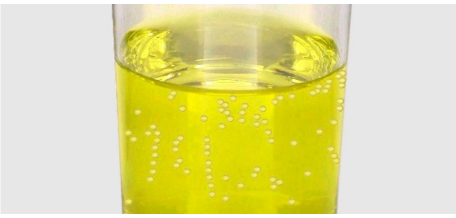 | 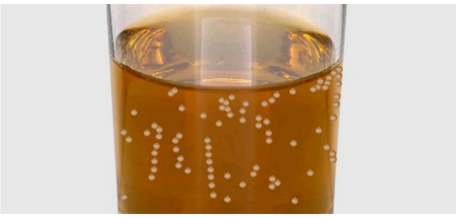 |
| Width 3 | 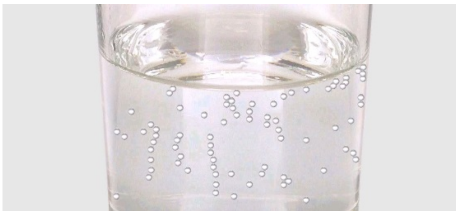 | 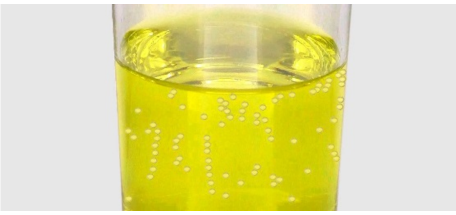 | 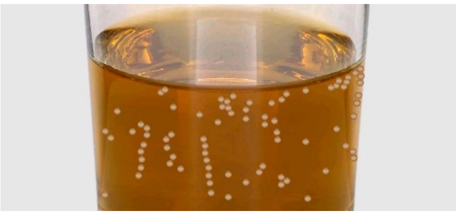 |
| Width 4 | 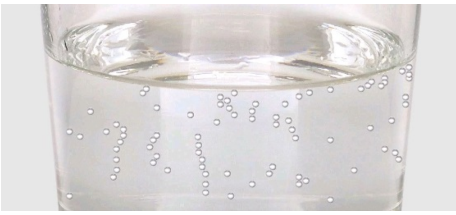 | 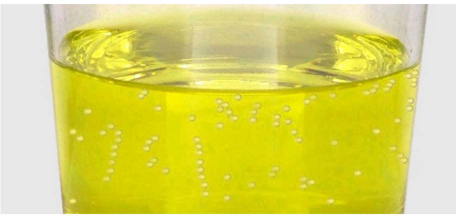 | 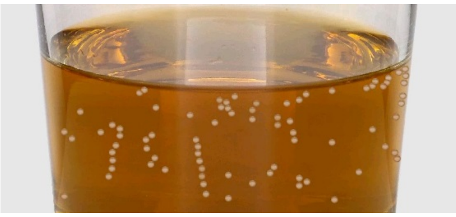 |

**Table S4.** Pictures of the neutral visual stimuli used in Experiment 2

The width of the glass for all neutral visual stimuli corresponded to the width 4.

| | Colorless | Yellow | Brown |
|---|---|---|---|
| No ice cubes | 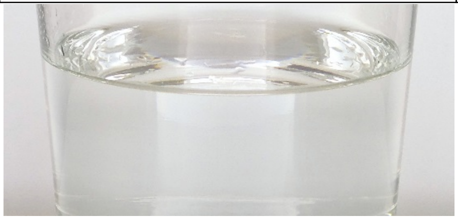 | 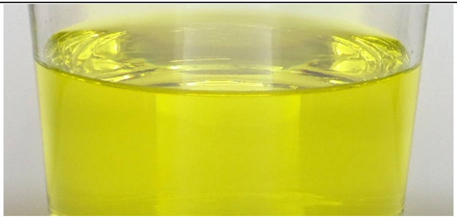 | 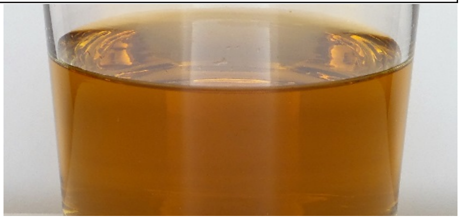 |
| Turbid | 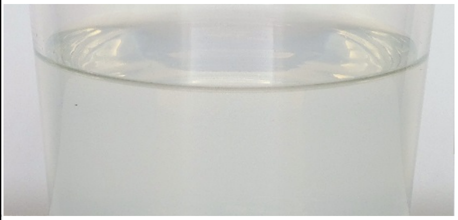 | 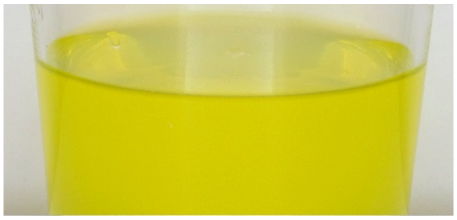 | 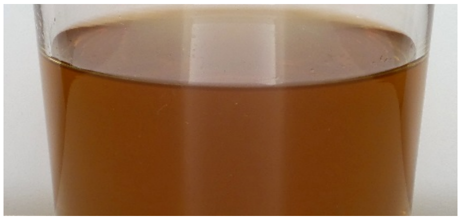 |
| Ice Cubes | 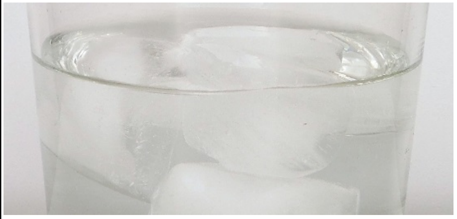 | 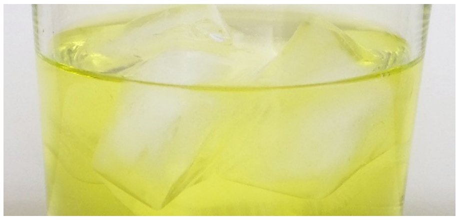 | 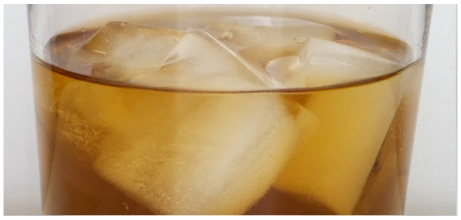 |
| Ice Cubes + Turbid | 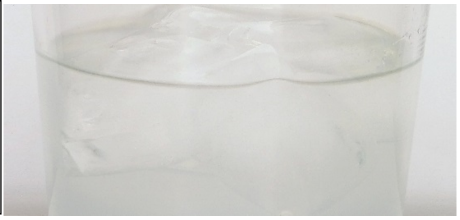 | 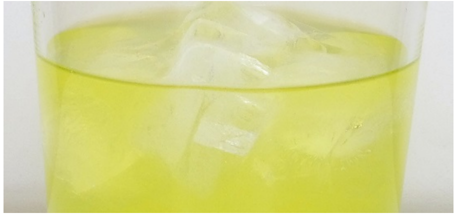 | 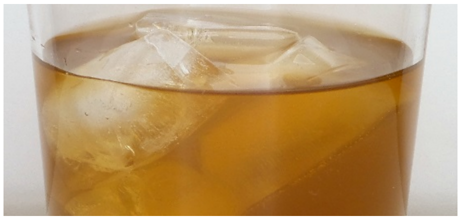 |

**Table S5.** Summary of the design for the GNAT blocks used in Experiment 2.

| | | | | |
|---|---|---|---|---|
| 1 | Single task (practice) | Small bubbles | Big bubbles | 24 |
| 2 | Single task (practice) | High-pitched | Low-pitched | 24 |
| 3 | Single task (practice) | Big bubbles | Small bubbles | 24 |
| 4 | Single task (practice) | Low-pitched | High-pitched | 24 |
| 5 | 1st combined task (practice) | Small bubbles+high-pitched | Big bubbles+low-pitched | 24 |
| 6 | 1st critical combined task | Small bubbles+high-pitched | Big bubbles+low-pitched | 72 |
| 7 | 2nd combined task (practice) | Small bubbles+low-pitched | Big bubbles+high-pitched | 24 |
| 8 | 2nd critical combined task | Small bubbles+low-pitched | Big bubbles+high-pitched | 72 |
| 9 | 3rd combined task (practice) | Big bubbles+high-pitched | Small bubbles+low-pitched | 24 |
| 10 | 3rd critical combined task | Big bubbles+high-pitched | Small bubbles+low-pitched | 72 |
| 11 | 4th combined task (practice) | Big bubbles+low-pitched | Small bubbles+high-pitched | 24 |
| 12 | 4th critical combined task | Big bubbles+low-pitched | Small bubbles+high-pitched | 72 |

## Experiment 3:

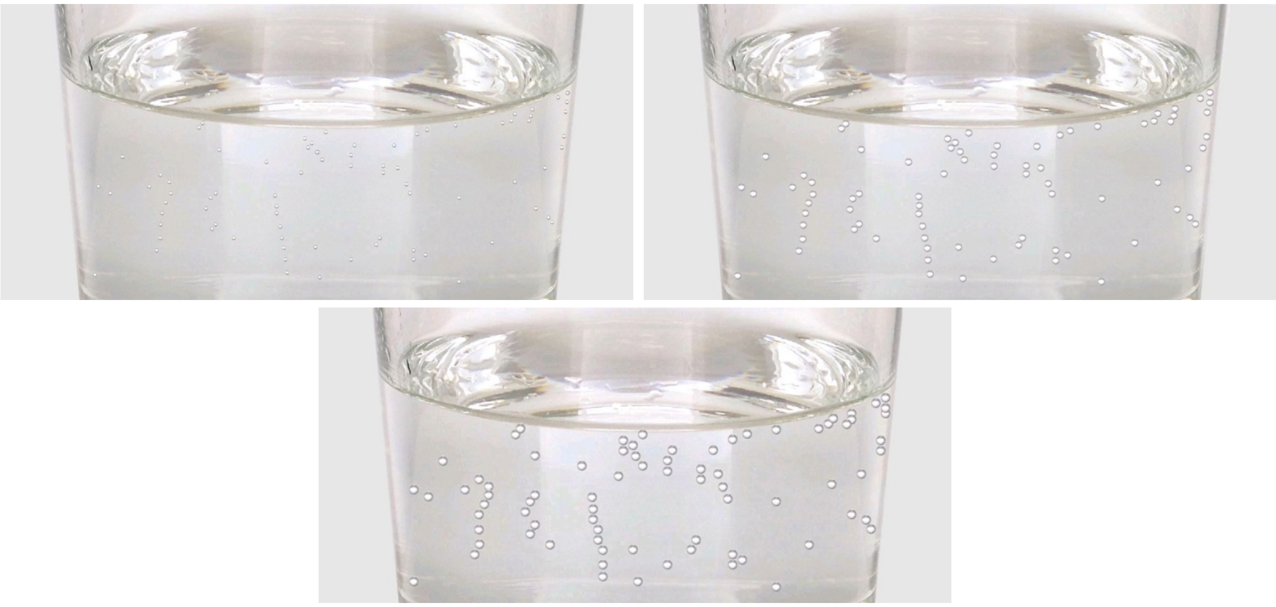

**Figure S1.** Pictures of the three visual stimuli used in Experiments 3a and 3b, with different bubbles' sizes.
